# The Right Man for the Right Place!

**Published:** 1861

**Authors:** 


### THE RIGHT MAN FOR THE RIGHT PLACE!

"The right man for the right place," is the cry of the hour; and a very good one it is too.—*North British Review.*

The war of the Crimea taught the British Government a lesson of great and permanent value. It saw one of its best equipped, most thoroughly provisioned, and apparently most formidable armies, gradually brought into a state of comparative inefficiency, and almost helplessness, through a series of blunders the result of official incompetency. The troops, half clothed, perished with cold, while ship-loads of warm clothing were within their sight; they toiled incessantly in snow and frost, half-famished, while the luxuries of living filled the commissariat; they perished of fever, dysentery, and cholera, in unprovided hospitals, without medical treatment, while hospital stores crowded the apothecary's department. The heart of the English people was touched by the tales of suffering, misery, and death, which came from their friends and brethren, and soon was heard the cry of popular indignation from one end of the realm to the other, and the imperious demand: THE RIGHT MAN FOR THE RIGHT PLACE!

If the British Government, with all its experience, could commit so grave a mistake as to instal unqualified persons in high or responsible positions in the perilous times of war, how infinitely greater is our danger of falling into this irremediable error? Our General and State Governments are profoundly ignorant of the art of war; they know nothing of its exigencies, its requirements, its laws, or its spirit. The former has for years had but a handful of half-famished troops on its borders, guarding the settler from the attacks of savages; hundreds of half-finished fortifications falling to decay, for lack of interest in their completion; and a school for military training, the educational nursling of the sons of a few Congressmen; while the latter have allowed their military laws to become a dead letter, or have abolished them altogether. Among the people at large the sword has literally been beaten into the ploughshare, and the spear into the pruning-hook; and their entire devotion to the arts of peace, and of a Christian civilization, might have been taken as a proof that they would learn war no more.

But suddenly the General Government summons from the States a vast army, and demands its immediate rendezvous at the National Capital; the State Governments respond with alacrity, and here our short-comings first appear. The State military offices have been filled without regard to the qualifications of the candidates. Too often the incumbent has not only been utterly ignorant of his duties during his entire term, but what is especially to be deplored, incompetent to their proper fulfillment, should the emergency occur. The general complaint that now reaches us from every encampment proves but too conclusively the truth of our remarks. The military spirit of the people being aroused, the supply of troops greatly exceeds the demand. The preparation of the outfits of this army opens a vast system of stock-jobbing, which is eagerly welcomed by the thousands who are ready to "turn a penny" by any new adventure. The weak and imbecile officials readily become the tools of designing men, and exercise the functions of their office without discretion, or for mercenary purposes. As a consequence, the troops have been clothed with garments that would shame a convict, and have been entertained with food that rendered their summons to mess more to be dreaded than an order to prepare for battle.

With such a class of officers to commence the work of organizing this immense army, it is not strange that many of the most important positions have been filled by men wholly unfit for the stations to which they have attained. At every place of rendezvous this fact is apparent on the most superficial examination, and at length it has been exhibited on the field of conflict. If these fatal errors in our army organization are not remedied in time, disasters will be multiplied, and ultimate defeat is not an improbability. But it augurs well for the intelligence of our people, and the final success of our Government, that these defects are already noticed, and are eliciting the watchword of reform—THE RIGHT MAN FOR THE RIGHT PLACE!

The pertinent inquiry will arise in the mind of every patriotic physician, who has also the honor of his profession at heart—Is the medical profession of the loyal States properly represented in this great uprising of the people for the maintenance of our national Government? The simple truth is, the medical profession has as yet not a proper representation or influence in this movement. Medical men wholly unqualified for their positions have, unfortunately, too often been already installed in important offices, from which emanate other appointments of the same low grade of qualification. In one State the question was asked by a leading paper, Who is our Surgeon-General? He was at length found, and proved to be a quack! It is not doubtful what will be the character of his subordinate officers. In another State, a surgeon, the brightest ornament of his profession, and who has a national reputation as an author, desiring to contribute his part to the good cause, early applied to the Surgeon-General of the State where he resided for the position of Medical Inspector at one of the rendezvous. He was, however, informed, by a communication from the Surgeon-General, that *no such office existed.* A few days after, the official bulletin announced the appointment, as MEDICAL INSPECTOR, at the very same rendezvous, and by the same Surgean-General, of a man of universally acknowledged incompetency. This fact has since been proved by the re-inspection ordered by the military authorities at Washington, of the troops which he had passed, and the discharge of large numbers as unfit for service.

The State Boards of Medical Examiners have proved, in many instances, either negligent, or culpably ignorant of their duties. We may estimate by hundreds the numbers of unqualified persons who have received the endorsement of these bodies, as capable Surgeons and Assisstant-Surgeons to regiments. Indeed, these examinations have in some cases been so conducted as to prove the merest farce. Irregular practitioners, "retired physicians," disabled "political doctors," physicians unable to obtain a livelihood in civil practice from sheer incapacity, have emerged from the "Green Room" full-fledged Army Surgeons. The result of this official ignorance is now apparent; the Secretary of War has recently called the attention of the Surgeon-General of the United States to the reported incapacity of regimental surgeons of the volunteer forces at Washington, and directed a re-examination, with a view to the dismissal of those found incompetent.

In the present number will be found the Plan of Organization of the Sanitary Commission, with the names of the members. A more important commission never was organized in this country, and it reflects most creditably upon the intelligence of our highest authorities, and their disinterested zeal in behalf of the welfare of our citizen soldiers. The investigations which this commission proposes to pursue, and the defects which it will aim to remedy, are of vital interest to the army, and involve, to a certain extent, the final issue of the struggle. The medical profession have a deep interest in the success of this commission, for it is in its incipiency, its duties, and its construction, a medical commission. It is, we believe, the first instance in which our Government has recognised a body as advisory in matters of a sanitary and purely medical character, independently of the medical department of the army. Our profession would gladly see this organization a permanent one. But its success depends upon the efficiency of the individual members of the commission. Distinguished as are the members of this body, and competent as they are to cope with the responsible duties which they have patriotically assumed, the medical profession will regret that the chivalrous State of Rhode Island could not have been represented by her distinguished Sanitarian, Dr. Edwin M. Snow; that New York could not add to its councils the knowledge of its wisest public Hygienist, Dr. John H. Griscom; and that Pennsylvania could not contribute the ripe experience of its practical Health Officer, Dr. Wilson Jewell. The names of many other gentlemen suggest themselves, whose life-long studies have eminently fitted them for the researches of this commission.

But we forbear to pursue this subject further. We have made these remarks in no captious or fault-finding spirit, but with a strong desire to see errors corrected, and evils removed, which if allowed will in the end prove dangerous if not disastrous. Especially do we desire to see the medical department of the volunteer army elevated above the low level of partisanship and favoritism. We trust that the re-examination of the regimental surgeons at the Seat of Government will be rigid, and that the service will be thoroughbly sifted of its unqualified medical officers. Let the popular feeling, which demands that incompetent commissioned officers of the line shall retire, or fall back into the ranks, be extended to the medical department, in all its branches, whether State or National. Let the motto of both Government and people in this struggle be:—THE RIGHT MAN FOR THE RIGHT PLACE!!

